# Supplementary material for: Personalized mapping of body homeostasis using whole-body PET connectomics and routine FDG PET imaging
Source: Commun Med (Lond). 2026 Mar 27;6:294. doi: 10.1038/s43856-026-01549-y (PMC13195144; doi:10.1038/s43856-026-01549-y)
Supplement: Supplementary file 2 — Supplemental Information [file 43856_2026_1549_MOESM2_ESM.pdf]

Personalized Mapping of Body Homeostasis Using Whole-Body PET Connectomics and Routine FDG PET Imaging - *Supplementary Materials*

Aldric Labarthe[1,2,a], Suzanne Varet[3], Laurent Savale[4,5,6], David Montani[4,5,6], Marc Humbert[4,5,6], Sylvain Faure[3,7], and Florent L. Besson[4,8,9,b]

[a]aldric.labarthe@ens-paris-saclay.fr (corresponding author)
[b]florent.besson@aphp.fr (corresponding author)

A Supplementary Method: Compression algorithm

As usual probability distances are often $O(n^2)$ with n the number of voxels for each organ, and as each organ is often made of millions of voxels, we had to develop an algorithm to reduce the voxels vectors.

One natural idea would be to downsample the SUV vector (the SUV vector is the vector associated with voxels: to each voxel - a 3d point - is associated a value, a SUV score) using a probabilistic method. While being cheap, as we want to assess interactions between organs, we cannot downsample SUV distributions with an algorithm that assumes SUV distributions to be independent. More, if the original vector is very large, downsampling will heavily affect tails, which can bias our probability distance computation step.

We therefore developed an algorithm that is interpretable and that consistently performs better on organs that are less than 2 millions voxels. The algorithm is a variation on the usual design of iterative point cloud reduction using graph-based exponential similarity.

We start from a set of N points $\{\mathbf{x}_i\}_{i=1}^N$ in $\mathbb{R}^d$, each endowed with a scalar activation a_i. Our goal is to iteratively merge highly similar points until no further merges remain, yielding a compact representation of the original cloud. At each iteration, we:

1. Construct a k-nearest-neighbour graph: using a spatial index (e.g. a k-d tree), we connect each point i to its k closest neighbours in Euclidean position.

2. Compute a combined similarity: for any pair of nodes i, j, we measure: $d_{\text{pos}} = \|\mathbf{x}_i - \mathbf{x}_j\|_2, d_{\text{act}} = |a_i - a_j|$, and convert each into a Gaussian kernel,

   $$s_{\text{pos}} = \exp\bigl(-d_{\text{pos}}^2/2\sigma_{\text{pos}}^2\bigr), \quad s_{\text{act}} = \exp\bigl(-d_{\text{act}}^2/2\sigma_{\text{act}}^2\bigr).$$

   These are then blended by an activation weight ω,

   $$s_{ij} = (1-\omega)\, s_{\text{pos}} + \omega\, s_{\text{act}}.$$

3. Select and merge similar pairs: we scan all edges (i, j) whose weight s_{ij} exceeds a threshold τ, sort them by decreasing similarity, and then—without reusing any node—merge each surviving pair. A merge replaces i, j by a new node whose position and activation are either the (unweighted) averages or the weights-averaged values,

   $$\mathbf{x}_{\text{new}} = \frac{w_i \mathbf{x}_i + w_j \mathbf{x}_j}{w_i + w_j}, \quad a_{\text{new}} = \frac{w_i a_i + w_j a_j}{w_i + w_j},$$

   with w_i, w_j the previous node weights (initially all 1). All edges to i or j are reconnected to the new node, with weights recomputed by the same combined-similarity formula.

4. Repeat until convergence: We continue up to a maximum iteration count T, or stop earlier if no edges exceed τ, or if the total node count remains unchanged for a prescribed number of steps.

The parameters ω and k are determined in using a trial-and-error method monitored by the resulting Wasserstein distance between the compressed distribution and the original one (we select parameters that minimize it). k is set to $\frac{1}{2}\sqrt{n}$ and ω starts at .7 and is increased by .02 at each time that we do not find any possible merge to perform. The similarity threshold is set to .95. These decreasing rules have been enforced as the algorithm can struggle if the organ is dispersed across the body (like the

fat is): with this addition, the algorithm will always prioritise similarity in SUV activation rather than spatial similarity, which is consistent as its function is to compress the SUV distribution vector.

To ensure reproducibility and scale-invariance, the Gaussian kernel widths are computed adaptively for each organ:

- σ_{pos} is set to the **median** distance of all nodes to their k-th nearest neighbor, ensuring the spatial kernel adapts to the local point density of the organ.
- σ_{act} is set to the standard deviation of the organ's SUV distribution ($\sigma_{\text{act}} = \text{std}(\text{SUV})$), normalizing the intensity differences.

Algorithm 1 Iterative point-cloud reduction with combined positional & activation similarity

Require: $\{\mathbf{x}_i, a_i\}_{i=1}^{N}$, degree k, thresholds τ, T, kernels $\sigma_{\text{pos}}, \sigma_{\text{act}}$, weight ω
Ensure: Reduced set $\{\mathbf{x}, a\}$ and final graph G
Build k-NN graph G on positions $\{\mathbf{x}_i\}$
for $t = 1$ to T **do**
  Compute all edge-weights s_{ij} by combining spatial and activation Gaussians
  Let $E' = \{(i, j) \mid s_{ij} \geq \tau\}$, sorted descending
  if E' is empty **then**
    return current nodes
  end if
  Initialize set $\mathscr{M} \leftarrow \emptyset$
  for each $(i, j) \in E'$ **do**
    if $i, j \notin \mathscr{M}$ **then**
      Merge $i, j \to$ new via weighted average; add $\{i, j\}$ to $\mathscr{M}$
    end if
  end for
  if no merges occurred **then**
    return current nodes
  end if
end for
return current nodes

In this scheme, the parameter k controls local connectivity, τ sets the minimum similarity to merge, and $\omega \in [0, 1]$ balances the influence of spatial versus activation differences. By merging multiple disjoint pairs per iteration and recomputing similarities on the fly, we achieve rapid reduction in point count while preserving both spatial structure and activation patterns. At the end, our algorithm returns three vectors: an activation vector, a voxel vector, and a weight vector. The weight vectors enable us to keep track of merged nodes: if several nodes have be merged into one, this node should be more important in the final distribution.

Using these weighted distributions, we can compute the energy distance:

$$\mathscr{E}_w(P, Q) = 2\sum_{i=1}^{n}\sum_{j=1}^{m} w_i v_j \|\mathbf{x}_i - \mathbf{y}_j\| - \sum_{i=1}^{n}\sum_{i'=1}^{n} w_i w_{i'} \|\mathbf{x}_i - \mathbf{x}_{i'}\| - \sum_{j=1}^{m}\sum_{j'=1}^{m} v_j v_{j'} \|\mathbf{y}_j - \mathbf{y}_{j'}\|,$$

We provide a benchmark of our algorithm with respect to the usual downsampling technique (Uniform Random Sampling, the Monte Carlo approach where voxels are selected with equal probability) in Figure 1. Overall, we achieve better performance in almost all organs (except for those which are not spatially concentrated like the skeleton muscles). Even more, the Wasserstein distance does not highlight the preservation of spatial consistency achieved by our method.

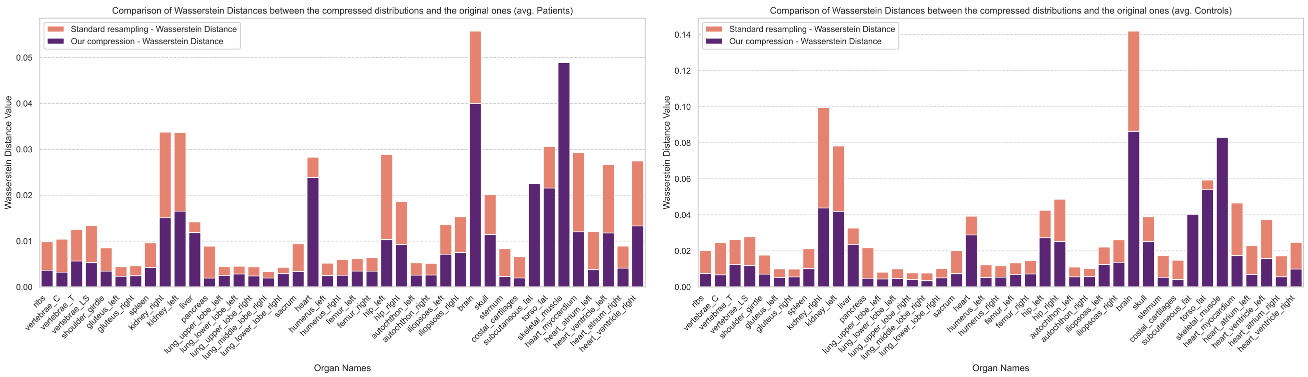

(a) Patients

(b) Controls

Figure 1. Benchmarking of the compression algorithm. Comparison of the proposed compression method versus uniform random sampling for (a) Patients and (b) Controls. Lower Wasserstein distance indicate better preservation.

B Supplementary Result: Classification performance using logistic regression

To establish robust classification results to our small sample size, we used a regularized logistic regression framework. This approach provides a transparent benchmark to evaluate whether the predictive patterns captured by the more complex Graph Convolutional Network (GCN) could be replicated by lower-capacity models. To ensure the generalizability of our findings, we conducted a systematic hyperparameter optimization via grid search, evaluating three distinct regularization regimes: L1 (Lasso), L2 (Ridge), and unpenalized.

The validation strategy employed a repeated stratified k-fold cross-validation ($k = 3$, 50 repetitions), ensuring that each fold maintained the original class distribution while providing a stable estimate of the model's performance and variance. Performance was quantified using balanced accuracy to account for class imbalance. Finally, to rigorously test the null hypothesis—that the model's performance could be achieved by chance—we performed permutation testing ($n = 1000$). Table summarizes the performance metrics for each regularization strategy.

Table 1. Classification performance across different regularization regimes for the logistic regression baseline. Results represent the mean and standard deviation across 50 repetitions of 3-fold stratified cross-validation. p-values are computed with permutation testing ($n = 1000$).

Penalty	Optimal C	Mean Balanced Accuracy ($\pm$ SD)	p-value
L_1 (Lasso)	0.1091	$73.29\% \pm 7.97\%$	0.002
L_2 (Ridge)	0.1091	$73.12\% \pm 8.37\%$	0.001
None	N/A	$74.11\% \pm 8.09\%$	0.001

To interpret the learned decision boundaries, model coefficients were extracted from the optimized logistic regression configurations (Figure 2). These weights were mapped back into a symmetric ROI-to-ROI space to visualize the connectivity patterns driving the classification. By comparing L1-penalized (sparse) and L2-penalized (distributed) weight matrices, we identified consistent pairwise features that distinguish PAH patients from controls. Notably, the most discriminative connections were localized primarily around the right heart chambers, with marked emphasis on the right-heart–spleen and pancreas–spleen axes. This comparative approach demonstrates that the identified biomarkers are robust to the choice of regularization and model architecture. Furthermore, the alignment between these linear decision boundaries and our GCN saliency maps suggests that the primary biological drivers of PAH classification are characterized by strong, consistent feature interactions that remain detectable across varying levels of model complexity.

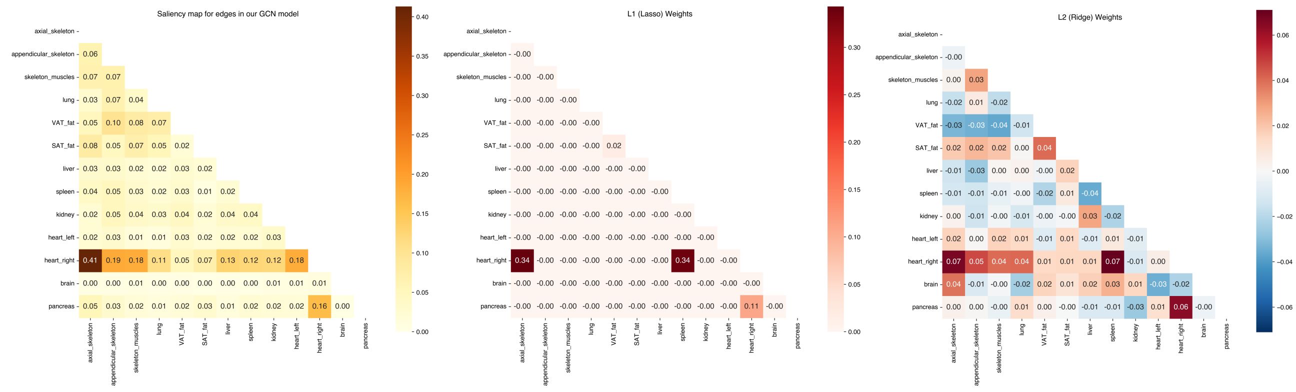

(a) GCN Saliency map **(b)** Logistic-L1 coefficients **(c)** Logistic-L2 coefficients

Figure 2. Comparison of feature importance across classification models. (a) Edge saliency map derived from the Graph Convolutional Network (GCN). (b) Coefficients from L1-regularized logistic regression. (c) Coefficients from L2-regularized logistic regression. Darker colors indicate a higher importance of the pairwise link for distinguishing Patients from Controls ($\alpha = 1$).

C Supplementary Note: Cohort Demographics

Table 2. Demographic, biologic, and hemodynamic characteristics of the study cohort.

	**Controls** ($N = 46$)				**Patients** ($N = 22$)			
**Characteristic**	N	**Min**	**Mean**	**Max**	N	**Min**	**Mean**	**Max**
Sex (Female)	16	-	-	-	13	-	-	-
Age (years)	46	29	52.4	67	22	20	46.4	62
Weight (kg)	46	48	76.5	120	22	37	68.4	100
Injected Activity (MBq)	46	149	267.1	383	22	139	242.8	379
Glycemia (mmol/L)	46	3.9	5.2	7.0	22	3.9	5.1	6.2
Hemodynamic Parameters (Patients only)								
RAP (mmHg)	-	-	-	-	18	3.0	7.9	18.0
mPAP (mmHg)	-	-	-	-	18	39.0	59.3	91.0
PAWP (mmHg)	-	-	-	-	15	7.0	11.2	19.0
CO (L/min)	-	-	-	-	16	2.87	5.08	6.67
CI (L/min/m^2)	-	-	-	-	18	1.50	2.74	3.60
PVR (WU)	-	-	-	-	18	3.28	11.97	26.30
TPR (WU)	-	-	-	-	6	8.49	9.62	12.27

Abbreviations: RAP: Right Atrial Pressure (POD); mPAP: Mean Pulmonary Arterial Pressure; PAWP: Pulmonary Artery Wedge Pressure (PAPO); CO: Cardiac Output (DC); CI: Cardiac Index; PVR: Pulmonary Vascular Resistance (RVP); TPR: Total Pulmonary Resistance; WU: Wood Units.
