# Supplementary material for: Personalized mapping of body homeostasis using whole-body PET connectomics and routine FDG PET imaging
Source: Commun Med (Lond). 2026 Mar 27;6:294. doi: 10.1038/s43856-026-01549-y (PMC13195144; doi:10.1038/s43856-026-01549-y)
Supplement: Supplementary file 3 — Description of Additional Supplementary files [file 43856_2026_1549_MOESM3_ESM.docx]

**Description of Additional Supplementary Files**

File name: Supplementary Data 1

Description: t-test results for figure 6
